# Role of Ovarian Hormones in the Modulation of Sleep in Females Across the Adult Lifespan

**DOI:** 10.1210/endocr/bqaa128

**Published:** 2020-07-31

**Authors:** Alana M C Brown, Nicole J Gervais

**Affiliations:** Department of Psychology, University of Toronto, Toronto, Ontario, Canada

**Keywords:** menopause, menstrual cycle, sleep disorders, fragmented sleep, spindles, slow-wave sleep

## Abstract

Ovarian hormones, including 17β-estradiol, are implicated in numerous physiological processes, including sleep. Beginning at puberty, girls report more sleep complaints than boys, which is maintained throughout the reproductive life stage. Sleep problems are exacerbated during the menopausal transition, evidenced by greater risk for sleep disorders. There is emerging evidence that menopause-associated hormone loss contributes to this elevated risk, but age is also an important factor. The extent to which menopause-associated sleep disturbance persists into postmenopause above and beyond the effects of age remains unknown. Untreated sleep disturbances have important implications for cognitive health, as they are emerging as risk factors for dementia. Given that sleep loss impairs memory, an important knowledge gap concerns the role played by menopause-associated hormone loss in exacerbating sleep disturbance and, ultimately, cognitive function in aging women. In this review, we take a translational approach to illustrate the contribution of ovarian hormones in maintaining the sleep–wake cycle in younger and middle-aged females, with evidence implicating 17β-estradiol in supporting the memory-promoting effects of sleep. Sleep physiology is briefly reviewed before turning to behavioral and neural evidence from young females linking 17β-estradiol to sleep–wake cycle maintenance. Implications of menopause-associated 17β-estradiol loss is also reviewed before discussing how ovarian hormones may support the memory-promoting effects of sleep, and why menopause may exacerbate pathological aging via effects on sleep. While still in its infancy, this research area offers a new sex-based perspective on aging research, with a focus on a modifiable risk factor for pathological aging.

Women spend approximately one-third of their lifespan in postmenopause, a hormone-deprived state that typically begins ~50 years of age. The transition to menopause (ie, perimenopause) is characterized by symptoms including sleep disturbance. While menopausal symptoms can persist 10+ years beyond the last menstrual period (1), the majority of research has focused on sleep complaints during perimenopause and early postmenopause. This is unfortunate, as sleep disturbance increases with age, with 22% to 39% of people aged 47 to 69 years (2) and >50% of people 65+ years (3) reporting sleep complaints. Additionally, there is emerging evidence for a role of sleep disorders in exacerbating risk for dementia, including Alzheimer’s disease (AD). The prevalence of sleep disorders increases following menopause, with some evidence suggesting this is independent of aging. The extent to which ovarian hormone deprivation contributes to sleep disturbance in aging postmenopausal women remains elusive.

This mini-review draws from human and animal literature to present our current understanding of the role of ovarian hormones in regulating sleep and cognitive health across the adult lifespan, with a discussion on long-term consequences of menopause-associated hormone loss. We review the clinical literature spanning the reproductive years, before discussing the consequences of menopause, and potential benefits of hormone therapy (HT) use. We have emphasized the role of 17β-estradiol (E2), the most common (across species) and bioactive estrogen. E2 is also increasingly used in HT, and so is receiving greater research focus. Progesterone (P) is understudied, therefore a more limited discussion of this hormone is provided.

## Overview of Physiological Sleep Processes

**Figure 1.**
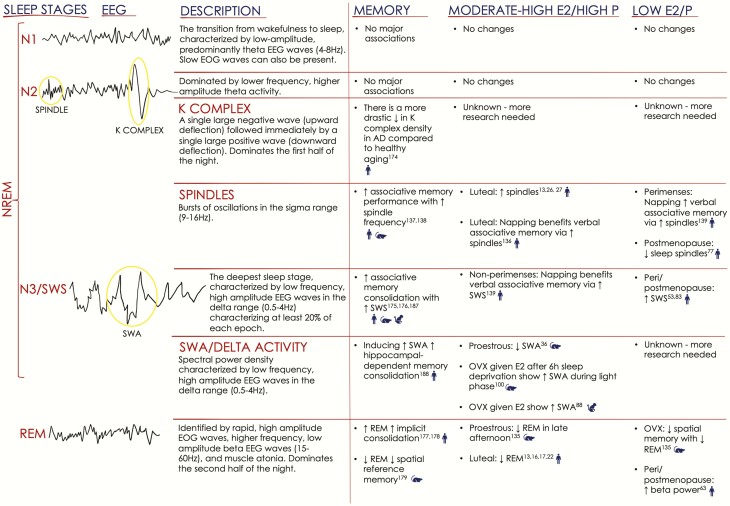
Summary of sleep physiology/stages, associations with memory, and changes related to hormonal milieu. Illustrations on the left depict EEG activity associated with each of the four stages of sleep (American Academy of Sleep Medicine (4)), and include definitions. Note that sleep staging is also conducted using concurrent EMG and EOG recordings. The right panel summarizes memory and hormone findings for rodents, nonhuman primates, and humans, denoted with blue symbols. AD = Alzheimer’s disease; E2 = 17β-estradiol; EEG = electroencephalography; EMG = electromyography; EOG = electrooculography; NREM N1-3= nonrapid eye movement stages 1-3; OVX = ovariectomized; P = progesterone; SWA = slow-wave activity; SWS = slow-wave sleep; REM= rapid eye movement.

Sleep is quantified using polysomnography (PSG), a combination of electroencephalography (EEG), electromyography (EMG), and electrooculography (EOG), which is used to classify sleep stages, including rapid eye movement (REM) and non-REM (NREM, further subdivided into three stages, N1-N3) sleep. Additionally, PSG permits the measurement of other parameters, including total sleep time, sleep latency (duration to first sleep stage), sleep efficiency, and frequency of awakenings and arousals (transient increases in cortical activity during sleep, Fig. 1) (4). EEG activity during sleep is characterized by synchronous and desynchronous activity between regions such as the hippocampus, thalamus, and frontal cortex. N3 is the deepest stage and is characterized by slow-wave EEG activity reflecting synchronous activity within the frontal cortex. Humans tend to cycle through these stages every 1.5 hours, and sufficient sleep (7-9 hours) typically includes 3 to 5 cycles/night (5).

Nonhuman animals commonly used in sleep research include mice and rats, both of which are nocturnal and polyphasic, with some sleep bouts occurring during their active (ie, dark) phase. Despite differences in their sleep timing compared with humans, rodent studies provide valuable insight into mechanisms underlying the sleep-wake cycle that are ubiquitous across mammals.

The timing and duration of sleep is regulated by 2 processes: homeostasis and circadian rhythms. Homeostasis is observed through sleep pressure, which gradually increases in proportion to time awake, and decreases with time asleep (6). Adenosine levels follow the same pattern and serve as an index of sleep drive/need (7). Homeostatic regulation of the sleep–wake cycle is observable under sleep deprivation/restriction procedures, as extended time awake elevates sleep pressure. The amount of time spent awake is typically proportional to time spent in subsequent N3 (ie, slow-wave sleep, SWS) during recovery sleep.

Circadian rhythms are responsible for synchronizing our sleep–wake cycle to a ~24-hour clock (6). Circadian control of sleep is regulated by the suprachiasmatic nucleus (SCN) of the hypothalamus (8). This structure lies directly above the optic chiasm, allowing direct input from afferent photosensitive neurons, whose activation promotes light-induced gene expression within the SCN that are entrained to the light/dark cycle. This master clock regulates a number of functions, including the release of melatonin from the pituitary gland. The secretion of this hormone is suppressed by daylight, and rising levels at night are involved in sleep initiation (9). As such, the SCN and circadian rhythmicity promote wake during daylight and, in collaboration with accumulating sleep pressure (via homeostasis), sleep following nightfall, approximately 15 to 17 hours after wake onset (6). The extent to which E2 directly modulates homeostatic and circadian processes is discussed in the “Role of E2 in homeostatic regulation of the sleep–wake cycle” and ‘Role of E2 in circadian regulation of the sleep–wake cycle.”

## Contributions of Ovarian Hormones to Sleep During Reproductive Years

**Figure 2.**
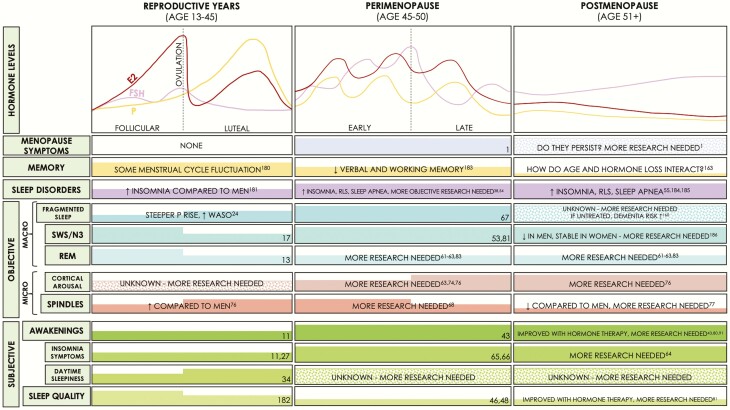
Summary of hormone levels across different reproductive states in women, and known effects on memory and sleep. Left panel summarizes findings in women of reproductive age (with normal ~28-day cycles), the middle panel for women in perimenopause, and the right panel for women in postmenopause. The top bar summarizes hormone levels during the 3 reproductive states, with menopausal symptoms, memory findings and sleep disorder prevalence underneath. Specific findings with respect to how sleep is measured are presented in the middle and lower bars. Thick colored bars within each cell indicate higher levels of symptoms/memory/sleep parameters, and colored dots indicate incomplete/unknown information. E2 = 17β-estradiol; EEG = electroencephalography; EMG = electromyography; EOG = electrooculography; FSH = follicle stimulating hormone; N3 = nonrapid eye movement stages 1-3; OVX = ovariectomized; P = Progesterone; SWA = slow-wave activity; SWS = slow-wave sleep; REM= rapid eye movement; RLS = restless legs syndrome; WASO = waking after sleep onset.

The menstrual cycle is ~28 days in length and consists of the follicular and luteal phases. Ovarian hormones (E2/P) are low during the early follicular phase, but E2 rises rapidly towards the end. The luteal phase is generally associated with moderate E2 and high P levels, which decrease prior to menses onset (Fig. 2, Reproductive Years) (10). Cross-sectional studies comparing women in follicular to luteal phase report no group differences in sleep latency, waking after sleep onset (WASO), and sleep efficiency (SE; % time asleep in bed) (11-14). Earlier REM onset (15), reduced REM (13, 16-20), and increased SWS (17) are observed during the luteal phase of some studies, whereas others report no differences (13, 21, 22) (Fig. 2; comprehensive reviews are provided by others (11, 12, 14, 23)). The rate of change rather than absolute hormone levels may alter sleep, as evidenced by the association between decreasing E2/P levels during the late luteal phase and reduced SWS (20). Further, increased WASO correlates positively with a steeper rise in P during the midluteal phase (24).

Microstructural measures are sensitive to menstrual phase (12, 13, 25, 26). Greater spindle activity is observed during the luteal than follicular phase (13, 26, 27), which persists until perimenopause (28). The underlying mechanisms have yet to be identified, but one possibility is that P modulates GABA_A_ receptors to increase spindle activity (13, 29). The hypnotic-sedative effects of P have been demonstrated in both cats (sex unspecified) (30), and postmenopausal women following frequent sleep disruptions (31). While these studies implicate P rather than E2 in promoting spindle activity and sleep more broadly, E2 may act indirectly via P, as higher E2 levels have been shown to increase P receptor levels (32).

Additional physiological factors sensitive to menstrual cycle phase may also contribute to the findings reported above. Fluctuations in REM duration are observed as a function of nighttime body temperature during the luteal, but not follicular phase (13, 16, 17, 33). Greater daytime sleepiness and nap SWS duration have also been shown during the luteal phase (34). This is consistent with findings showing increased daytime sleepiness/reduced alertness during the late-luteal phase, but only among women with elevated premenstrual symptoms (20). Since body temperature also has a diurnal rhythm, perhaps the menstrual cycle effects on sleep and temperature reflect the same underlying alterations in circadian rhythmicity.

In rodents, E2 promotes the consolidation of the sleep–wake cycle by facilitating wakefulness rather than improving sleep per se. The estrous cycle is 4 to 5 days in length and includes 4 phases (35). Proestrous is characterized by rapidly rising E2/P, which peak in the evening. This is followed by rapid decline in E2/P the following day (estrous). Moderate levels of these hormones are observed during the 2 remaining days (diestrous I/II). The evening of proestrous is associated with reduced spontaneous sleep (attenuated slow-wave activity (SWA or delta activity; 0.5-4 Hz) and REM, along with increased high-frequency cortical arousal and fragmentation) (36). This sleep suppression is not surprising, as this time frame is also marked by high levels of activity (37).

In sum, these studies implicate ovarian hormones in modulating the sleep–wake cycle during the reproductive years. However, sleep is not always promoted by elevated E2/P. In rodents, sleep is suppressed by the rapid rise of E2/P during proestrous, particularly during the active phase. In humans, rapidly increasing P during early luteal is associated with increased WASO, while declining levels of E2/P during late luteal are associated with reduced SWS. The luteal phase as a whole is associated with selective increases in SWS/spindles, but is also associated with reduced REM, and increased WASO/sleepiness. The extent to which these findings reflect absolute versus fluctuating levels of ovarian hormones needs further investigation.

## Contributions of Menopause-associated Hormone Changes to Sleep Disturbance

Perimenopause is characterized by gradually declining hormone levels (Fig. 2) and characteristic symptoms, including sleep disturbances, memory problems, mood changes, and hot flashes (HFs) (38, 39), along with increasing menstrual cycle length, and eventually irregular periods (for a more thorough description, see work from Harlow and colleagues (40)). HFs are characterized by intense sensations of heat, followed by sweating and skin vasodilation (39, 41). Hormone levels stabilize at postmenopause, which begins 12 months after the last menstrual period (40). Menopausal symptoms can continue for 10+ years (1), the consequences of which remain elusive. Importantly, optimal sleep maintains brain health, and untreated sleep disturbances can have important implications in aging postmenopausal women. What follows is a review of the literature on menopause-associated sleep disturbance and the role of ovarian hormone loss.

### Subjective sleep

During premenopause, 20% to 40% of women report sleep complaints, which increases to 40% to 60% during peri/postmenopause (42). Further, higher follicle-stimulating hormone (FSH)/lower estrone levels are also associated with greater severity of self-reported nighttime awakenings in peri/postmenopausal women (43) (but see (44)). Studies in perimenopausal women show that decreasing E2/increasing FSH are related to more awakenings (45), poorer sleep quality (46), and trouble sleeping (47). Declining E2 prior to perimenopause is also related to lower sleep quality (48). Additionally, surgical menopause is associated with more sleep complaints than natural menopause (49), further supporting the possibility that rapid changes in hormonal milieu contribute to sleep disturbance.

### Sleep disorders

Peri/postmenopause is associated with increased prevalence of insomnia (characterized by difficulty falling and/or staying asleep), restless leg syndrome (characterized by uncontrollable leg movement), and sleep apnea (characterized by interrupted breathing) (50-56). While age itself is a risk factor for sleep apnea (57), higher apnea–hypopnea index (number of apneas or hypopneas/hour) and lower arterial oxygen saturation are observed in postmenopausal than in premenopausal women even after controlling for age (53), indicating that hormone loss also contributes. Daytime sleepiness, which is a symptom of sleep disorders, is also higher among postmenopausal women (58). In peri/postmenopausal women with frequent self-reported sleep disturbances, periodic limb movements and apneas are the strongest predictors of diminished SE (55). Compared with natural menopause, surgical menopause is associated with more insomnia symptoms, reduced total sleep time and SE (50, 59, 60), and a 27% higher risk of sleep apnea (59). These findings support an important role of ovarian hormone deprivation in some sleep disorders above and beyond the contributions of aging. An important consideration relates to whether these conditions persist beyond midlife, as they are implicated in pathological aging (see “Interaction between ovarian hormones and sleep: implications for cognitive health and pathological aging”).

### Objective sleep

Paradoxically, peri/postmenopause are associated with increased SWS duration (46, 53, 61, 62) (but see (63)). While this certainly can reflect improved sleep, higher rates of sleep complaints/disorders in these women suggests this is unlikely. Additionally, peri/postmenopausal women report lower sleep satisfaction while also demonstrating increased SE and SWS (62). While some studies report no differences between pre- and peri/postmenopausal women on sleep macrostructure, including sleep latency, total sleep time, and SE (64-66), 1 prospective study showed increased fragmented sleep in women who transitioned from premenopause to peri/postmenopause 6 years later, even after controlling for HFs, depressive symptoms, and body mass index (67). Higher FSH levels have been associated with more WASO/awakenings/arousals in perimenopausal women, but only among those without insomnia (68). Increases in FSH were also positively associated with SWS (67). As discussed in “Role of E2 in homeostatic regulation of the sleep–wake cycle,” SWS duration is directly proportional to sleep need. Perhaps this increase in SWS reflects reduced sleep quality, or increased sleep fragmentation, especially considering that total sleep time is reliably unaffected (68). While the data presented above are certainly consistent with this interpretation, advancing age may also be implicated. Fragmented sleep does increase with age, but SWS tends to decrease, particularly in men (69). Thus, the observed findings might reflect changes specific to aging women that are due at least in part to ovarian hormone deprivation.

Co-occurring menopausal symptoms, including HFs, can contribute to greater sleep disturbance. HFs are reported by 60% to 90% of peri/postmenopausal women (70-72), with frequency correlating with greater sleep complaints (73), WASO, and reduced SE (41). Augmented HFs are observed in middle-aged women with insomnia, and are associated with more frequent awakenings (74). Thus, sleep disturbance experienced by perimenopausal women is likely due at least in part to HFs. Depressive symptoms, which are more common during peri/postmenopause, also contribute to sleep disturbance (75). Importantly, insomnia is also a symptom of major depression. Thus, the extent to which these disturbances occur as a direct result of E2 loss, independent of age and/or other symptoms/conditions, is not well established.

High cortical arousal within the beta frequency range (15-30 Hz) is indicative of less restful sleep, and has been observed in an older mixed-sex sample (76), and among perimenopausal women with insomnia (74). Higher beta power is observed in late peri/postmenopausal women than in pre- and early perimenopausal women, even after controlling for age, whereas delta power (0.5-4 Hz) is unaffected (63). This latter result is surprising given the reported increases in SWS in other studies. Thus, while peri/postmenopausal women spend more time in SWS, this may not reflect more intense sleep. These findings suggest that sleep is less restful in late peri/postmenopausal women.

Spindle density decreases in women with age, whereas it increases in men (77), implicating ovarian hormone loss in age-related EEG sleep physiology. While aging effects on sleep physiology are well understood (78, 79), limited attention has been paid to how menopause status, duration of hormone deprivation, and even HT use might alter sleep parameters in aging research. These important considerations can clarify how sleep disturbances manifest in aging women, particularly beyond the early postmenopausal years. The next section helps to clarify the role of ovarian hormones by making comparisons between groups that are hormone-deprived to those taking exogenous hormones matched by age.

### Hormone therapy

Hysterectomized postmenopausal women (47-65 years) taking unopposed transdermal E2 had fewer movement-related arousals during sleep and reported fewer HFs, sleep complaints, and headaches than nonusers (80). Peri/postmenopausal women taking E2 reported reduced insomnia symptoms and improved subjective sleep quality (81), although this may be restricted to women with HFs (82). Unopposed estrone is also associated with decreased awakenings and increased REM during perimenopause (45-55 years) (83), while estrone with micronized P (but not medroxyprogesterone acetate) increases SE and reduces WASO in postmenopausal women (45-65 years) (84). Strongest support for the positive effects of HT comes from a study using experimentally induced sleep disruptions (via nighttime blood draws). Older postmenopausal women (57-80 years) without HFs and >5 years from their last menstrual period taking either conjugated equine estrogen (CEE) or esterified estrogens (estrone/Equilin) for at least 2 years were compared with non-HT users. Sleep for the HT group was less disrupted by the manipulation, evidenced by reduced sleep latency, WASO, time awake, and increased SWS and SE (85). Together, these studies support subtle benefits of maintaining ovarian hormone levels on sleep macrostructure.

The major estrogen in CEE and esterified estrogens is estrone, which has a weaker affinity to estrogen receptors, and represents only ~4% of the estrogenic activity of E2 (86). The source, chemical structure, and composition of estrogens in commonly prescribed HTs vary in their pharmacokinetics and pharmacodynamics, which are further influenced by dosage and route of administration. Orally administered E2 results in low potency due to first-pass hepatic and intestinal metabolism into estrone and estrogen conjugates. This is not the case for nonoral routes, which bypass the intestines and liver. Thus, transdermal E2 has much greater bioavailability and potency than oral E2 (86). Despite the sedative-hypnotic effects of P (30), studies investigating HT fail to acknowledge its contribution. Studies clarifying the effects of E2 and P, including their dosages and routes of administration, are sorely needed, particularly in older postmenopausal women.

E2 promotes thermoregulation in addition to maintaining sleep in ovariectomized (OVX) animals. Inhibiting E2 synthesis in middle/older-aged female OVX marmosets increases facial temperature in response to a thermal challenge (87). Further, middle-aged OVX marmosets given E2 replacement demonstrate lower nighttime core body temperature, fewer nighttime arousals, and higher SWA (Fig. 1), signifying more intense and restorative sleep (88). At first glance, these findings seem contradictory to studies in women, which show that menopause (and therefore ovarian hormone deprivation) is associated with increased SWS. Importantly, these studies investigate sleep stage duration rather than SWA, a measurement of SWS intensity. EEG spectral power analyses provide better sleep depth/intensity quantification than visual sleep staging, and SWA or delta activity is often considered the primary indicator of homeostatic sleep regulation (89). While delta waves are unaffected by menopause status in women (63), the impact of E2 use during peri/postmenopause remains unexplored.

The potential benefit of E2/P in mitigating against menopause-associated sleep disorders has been largely ignored. Animal studies suggest that E2 reduces adverse effects of apnea/hypopneas (90), and 1 study in surgically menopausal women indicates that use of any type of HT reduces sleep apnea risk, while earlier menopause (and therefore increased time in hormone-deprived state) increases risk (59). Further, 1 month of E2+P was associated with fewer awakenings, reduced respiratory distress, and higher oxygen saturation among postmenopausal women (48-62 years) with sleep apnea (91). Finally, transdermal E2 improves sleep quality, reduces sleep latency, and decreases awakenings in postmenopausal women (47-65 years), especially those with insomnia (92, 93). Given that sleep apnea/insomnia are risk factors for Alzheimer’s disease (AD) (94, 95), attenuating risk at midlife can have important implications for women’s health. Thus, greater research attention paid to the potential role of E2/P in preventing sleep disorders at midlife is needed.

## Role of E2 in Homeostatic Regulation of the Sleep–Wake Cycle

To our knowledge, no study has specifically investigated whether ovarian hormones alter homeostatic control of sleep in women. Sex differences are observed in delta activity during rebound sleep, with more dramatic increases in young women than men, indicative of increased homeostatic sleep response (96). Interestingly, this sex difference is reversed in older age, with the quality of restorative sleep being better preserved among older men (97). The mechanism underlying this switch remains unknown, but it could involve change in menopause status in women. Future studies are needed to address the contribution of ovarian hormones to sleep pressure, and whether menopause-associated hormone loss attenuates this robust homeostatic response to sleep deprivation.

In female rodents, E2 promotes sleep following extended time awake. When the period of sleep deprivation is brief, the effects appear sensitive to circadian timing. After sleep deprivation, E2 consolidates the sleep–wake cycle by suppressing sleep (and increasing activity) during the dark phase, and promoting sleep during the light phase (98, 99). For example, OVX rats given E2 replacement and sleep deprived for 6 hours at the beginning of the light phase show increased REM and SWA during the remaining light phase (100). When recovery sleep occurs during the dark phase, E2 suppresses REM/NREM/SWA (98, 100). If sleep deprivation is sufficiently long (at least 12 hours), E2 appears to promote sleep, irrespective of circadian phase (99). Thus, unlike findings presented in “Contributions of ovarian hormones to sleep during reproductive years,” which demonstrated the wake-promoting effects of E2/P during the dark phase, the findings presented here complement studies in humans and indicate that E2 promotes sleep under sufficient sleep pressure or appropriate circadian timing. The sleep–wake cycle may be modulated by E2 at least in part via direct actions in brain regions that regulate sleep onset and maintenance. Estrogen receptors are expressed in the lateral hypothalamus, the medial preoptic area of the hypothalamus (MPOA) (101-104), and the ventral lateral preoptic nucleus (VLPO) (5, 105-111). Activation of MPOA neurons induces sleep onset, while VLPO neurons maintain sleep via inhibition of wake-active cells (112, 113). Damage to these regions decreases sleep duration without altering sleep timing, implicating these regions in homeostatic sleep drive and not circadian rhythmicity (114, 115). During the light phase, E2 decreases sleep-promoting lipocalin-prostaglandin D synthase expression and neural activity in the VLPO of OVX rats (116, 117). In addition, wake-promoting orexin neurons and receptors in the lateral hypothalamus, which also receive VLPO and MPOA input, are highly sensitive to E2 fluctuation in female rats, with high E2 increasing orexin expression in the hypothalamus and anterior pituitary of females during the evening of proestrus (118). The effect of E2 within these regions following sleep deprivation remains to be explored. Taken together, these findings suggest that high E2 levels inhibit spontaneous sleep in OVX rats, potentially promoting wakefulness to prepare for a more active dark phase (5, 119). An expanded discussion of the potential mechanisms of ovarian hormones on sleep exists elsewhere (5, 7, 78).

In sum, E2 modulates rebound sleep in humans and rodents. What remains unknown is whether advancing age alters the observed effects of E2. Additional studies that experimentally manipulate both hormone levels and sleep timing/duration while incorporating older rodents will advance our understanding of whether ovarian hormone deprivation exacerbates the effects of continued sleep disturbance in aging postmenopausal women. In addition to clarifying divergent effects between younger and older women, and between human and rodent studies, future research is needed to bridge the behavioral and molecular effects of E2.

## Role of E2 in Circadian Regulation of the Sleep–Wake Cycle

There are well-established cross-species sex differences in SCN morphology and activity (106, 120). In humans, the relative volume and length of the anterior–posterior axis of the SCN are larger in women (121), who also have more estrogen receptor α expression in this region than men (122). Circadian activity also varies by sex, as young/middle-aged women typically have an earlier chronotype (go to bed and wake up earlier) (106, 123), have higher peak levels of melatonin (124, 125), and lower temperature nadir than men (125).

There is some evidence that menopause alters circadian rhythmicity. Postmenopausal women (58-71 years) have lower nighttime melatonin concentrations and shorter secretion duration than perimenopausal women (43-51 years), suggesting that melatonin release may be suppressed by ovarian hormone deprivation and/or advancing age (126). This alteration in nighttime melatonin may lead to greater problems initiating or staying asleep, consistent with insomnia symptoms. Another important consideration is thermoregulation, which is critical for sleep maintenance. Menopause in humans is marked by HFs. As previously mentioned, E2 has been associated with lower nighttime core body temperature and more restorative sleep in middle/older-aged OVX marmosets (88). Since both body temperature and sleep are sensitive to circadian timing, these results suggest that ovarian hormones may play a crucial role in modulating circadian rhythmicity.

There is also evidence that ovarian hormones are sensitive to altered circadian rhythms, suggesting a bidirectional relationship. For example, 20+ months of rotating night shifts increases the risk of early menopause (before 45 years) (127). Shift work also increases the prevalence of menstrual disorders (128) and alters E2 level fluctuation (129) in women, implicating circadian rhythms in the physiological functions of E2.

In female rodents, the ability for E2 to promote sleep depends on circadian timing (see “Role of E2 in homeostatic regulation of the sleep–wake cycle”), and hypothalamic E2 activity is implicated in circadian rhythmicity. OVX reduces while E2 replacement increases melatonin binding sites and synthesis in the hypothalamus and medulla pons when samples are collected during the dark phase (130). Unfortunately, no known study has addressed whether E2 promotes melatonin production during the light phase. Greater consideration of circadian phase in rodent studies is needed to reconcile findings from animal and human literature.

In rodents, the ventral SCN responds to E2 replacement with increased neuronal firing (131) and enhanced transcription factor expression (132). Further, there are sex differences in the circadian patterns of neuropeptides in the lateral SCN, which are likely influenced by E2. For example, peak expression of the SCN signaling molecule vasoactive intestinal polypeptide-encoding gene, *vip*, occurs at the beginning of the dark phase in gonadally intact males, and during the light phase for females. OVX shifts the rhythm to be more male-like, whereas E2 administration returns the rhythm to the light phase (133). E2 also advances the expression of the Period Circadian Regulator 2 gene (*Per2*) to earlier (134), which may shift circadian timing to promote an earlier chronotype. It remains to be seen what impact menopause-associated E2 loss has on SCN functions in humans, and whether alterations may predispose women to sleep disorders, particularly insomnia.

## Interaction between Ovarian Hormones and Sleep: Implications for Cognitive Health and Pathological Aging

### Ovarian hormones modulate the memory-promoting effects of sleep

Despite the vast literature on memory-promoting sleep effects, few studies have addressed sex or hormonal milieu as potential modulators. In 1 study, REM was selectively reduced by placing rats on a platform in a water chamber for 72 hours. This platform permits rats to fall asleep, but prevents them from entering REM, as the associated atonia leads them to touch/fall into the water. Impaired spatial learning was demonstrated across groups, but was most pronounced in OVX rats (135), implicating E2/P in the learning-promoting effects of sleep in females. Human studies provide consistent support. For example, men and women in the midluteal, but not early follicular phase, benefited from postlearning napping (Fig. 1). E2 levels were positively associated with learning among women, and a learning-related increase in spindle activity was demonstrated only in men and midluteal women (136). Since spindles are important for memory consolidation (137, 138), these findings suggest that E2 plays a direct role in promoting sleep-dependent memory consolidation. Reduced postnap associative memory performance was also observed among women close to menses (–5 to +6 days around menses onset; perimenses; low E2/P), but not those outside the perimenses phase (>6 days from menses; nonperimenses). Associations with EEG events also differed across conditions, with performance correlating with spindles during perimenses and slow oscillations during nonperimenses (139). Thus, not only does hormonal milieu affect the ability to benefit from sleep, it also appears to influence which memory-supporting mechanisms are reinforced during sleep.

Ovarian hormones also modulate cognition across species, with the affected domains overlapping with those sensitive to sleep (66). There is also considerable overlap between the neuronal mechanisms impacted by sleep loss and those that support memory (140). For example, neurotransmitter systems implicated in sleep–wake cycle maintenance, including the cholinergic, glutamatergic, dopaminergic, and serotonergic systems, interact with E2 to modulate memory and memory-related brain regions in rodents (141-145). For example, ovarian hormone deprivation (via OVX or administration of the drug 4-vinylcyclohexene diepoxide) is associated with reduced levels of norepinephrine, serotonin metabolites, and amino acids (tryptophan and tyrosine) in the rat hippocampus (146). Melatonin is produced from serotonin via tryptophan (147); therefore, reduced tryptophan and/or serotonin levels may lower melatonin production. While speculative, perhaps melatonin levels are reduced following menopause due to altered serotonin availability. Altered synaptic plasticity may be another mechanism through which E2-sleep interactions promote memory (140). Given these findings, greater consideration of sex/hormonal milieu will improve identification of important mechanisms promoting the beneficial effects of sleep on memory.

### Ovarian hormone loss and pathological aging in women

Unfortunately, menopause-associated sleep disturbance and memory loss are typically studied in isolation. In addition to teasing apart the effects of aging from those of menopause-associated hormone loss, we also recommend cognitive measures be incorporated into future studies to identify the long-term consequences of sleep disruptions in postmenopausal women.

Women are more likely than men to develop AD, due in part to increased longevity (148). Older cognitively healthy women with elevated β-amyloid (characteristic of neurodegeneration) demonstrate more rapid cognitive decline than age-matched men with comparable β-amyloid levels (149). Among individuals with elevated genetic risk (ie, carriers of the ε4 allele of the apolipoprotein E gene), women are 43% more likely to develop mild cognitive impairment, ~4 times more likely to develop AD, and experience more rapid brain atrophy than male carriers (150). Animal studies corroborate these findings. Female transgenic AD mouse models have elevated β-amyloid accumulation (151), which accumulates at an earlier age than males (152). Together, these data indicate that female sex is a risk factor for AD. While speculative, perhaps menopause-associated sleep disturbance places women, particularly ε4 carriers, at higher risk.

In addition to supporting memory consolidation, sleep plays a critical role in the glymphatic system, a glial-dependent waste clearance pathway (153), involving the removal of toxins including AD-associated proteins (β-amyloid/tau). Toxins build up during wake and are eliminated during sleep, particularly SWS (154, 155), and acute sleep deprivation increases β-amyloid in mice (156) and young humans (157). Sleep fragmentation is a risk factor for AD (158), and emerges well before the clinical onset of AD (159). Sleep apnea, which is characterized by fragmented sleep, is also more common among patients with AD (160). However, the ε4 variant is also a risk factor for apnea (79), suggesting a bidirectional relationship between the 2. As discussed in “Role of E2 in homeostatic regulation of the sleep–wake cycle,” both fragmented sleep and sleep apnea prevalence increase in peri/postmenopausal women, and in younger women with surgical menopause (93). Insomnia, which is also higher among peri/postmenopausal women (64, 65), is also considered a risk factor for AD (161). If sleep disturbances emerge at midlife and remain untreated, they can adversely affect women’s brain health and cognitive functioning specifically, potentially elevating AD risk, particularly among ε4 carriers.

Estrogens are known to enhance cognition in females (162-166), yet few studies have focused on older females, including older postmenopausal women 60+ years. One important caveat related to the critical window hypothesis relates to the timing of treatment initiation, with many older studies initiating HT 10+ after last menstrual period, resulting in null findings or even adverse effects (167). Additionally, the effectiveness of HT may relate to the presence of ε4. For example, “oral estrogen” was found to reduce the risk of cognitive impairment among cognitively healthy older noncarriers (aged 65+ years), but had no effect on carriers (168). In another study, E2 use was associated with lower β-amyloid deposition among slightly younger postmenopausal (52-65 years) ε4 carriers (169). Taken together, these data suggest that while estrogens benefit cognition and reduce neurodegeneration, they may not prevent cognitive decline among ε4 carriers. It remains unknown whether E2 use alleviates sleep disturbance among older postmenopausal women, and to what extent ε4 status affects the pattern of results. Animal studies suggest that E2-induced synaptic sprouting and expression of apolipoprotein E messenger ribonucleic acids in rodents could support an E2–APOE interaction that warrants further investigation (170, 171).

Despite the elevated AD risk among older postmenopausal women, limited research has been devoted to understanding contributing factors. Genetic risk is obviously an important consideration, but additional factors, including sleep disruptions are becoming increasingly recognized, particularly as potential early markers. Postmenopausal women experience fragmented sleep and elevated risk for sleep conditions associated with AD, including apnea and insomnia. The contribution of ovarian hormone loss remains elusive. Future studies are needed to better understand the consequences of long-term ovarian hormone loss on sleep, including any interactions with neurotransmitter systems, whether poor sleep contributes to dementia risk and how early interventions targeting sleep and hormone levels may improve cognitive outcomes in aging women. Not only should future studies elucidate the effectiveness of E2/P in benefitting sleep at midlife, but also determine if they aid in prevention of pathological aging both among ε4 carriers and noncarriers.

## Conclusion

This mini-review highlights cross-species evidence supporting the involvement of ovarian hormones in maintaining the female sleep–wake cycle across the adult lifespan. In addition to neurological evidence implicating E2 in regulating the sleep-wake cycle, behavioral evidence suggests that E2 consolidates the cycle by promoting activity during the night and sleep during the day. In premenopausal women, the extant literature suggests subtle effects of menstrual cycle on sleep physiology. Peri/postmenopause is associated with elevated sleep complaints and disorders, but the sleep physiology literature is more limited, and suggests the effects are restricted to increased cortical arousal, SWS, sleep fragmentation, and spindle decline. Many of these studies are confounded by age. Stronger support comes from comparisons between HT users and non-users, which show improved self-reported sleep quality, reduced sleep latency and fragmentation, and decreased insomnia symptoms and sleep apnea risk. Correlational studies provide support for a direct role of E2 loss (or elevated FSH) on fragmented sleep.

Several gaps in knowledge remain, including the extent to which menopause-associated sleep disturbance continues beyond midlife, particularly for untreated sleep conditions, as they may predispose women to dementia, particularly among ε4 carriers. Additional gaps include the indirect effects of sleep disturbance on cognition at midlife, and the contributions of circadian rhythmicity and synaptic plasticity in menopause-associated sleep disturbance. Shift work is associated with early menopause (127) and can disrupt menstrual cycles in premenopausal women (128), but the long-term consequences on sleep and cognition are unknown. Importantly, early menopause has been associated with increased cardiovascular disease risk, as well as shorter life expectancy and cognitive decline (172).

Not only should greater research attention be placed on investigating sleep in older postmenopausal women, but researchers that study sleep and cognition need to place greater emphasis on addressing biological sex/hormonal milieu. This is particularly important in non-human animal studies, which frequently use male-only samples. Future studies of nonhuman animals considering time-of-day effects will clarify the precise functions of ovarian hormones in the brain while helping to identify long-term consequences of chronic sleep restriction.

In humans, the role of E2 in modulating sleep during other periods of hormonal flux, such as puberty and pregnancy, also warrants increased research attention to ensure that girls and women maintain optimal sleep. Addressing the potential benefits of specific HT types (eg, E2 with P) and the multifactorial nature of sleep disruption will help us to better characterize optimal healthy sleep, especially within the context of reduced memory and/or mood in everyday life. While our review provides convincing evidence for a role of ovarian hormones in sleep modulation across species, the literature is inconsistent. Studies that properly control for age, hormone regimen, and other relevant factors are needed before firm conclusions can be made.

Research currently suggests that 30% to 50% of medical residents in their final training year are “not at all” prepared to manage menopausal symptoms (173). This has obvious implications for peri/postmenopausal women seeking treatment for menopause symptoms and can have repercussions for their brain health years later. Ultimately, the effects of HT on sleep and brain function depend on initiation, duration of use, and type of HT, and the relevance of these variations on sleep, dementia, and cognitive aging remain to be confidently established. Sleep is altered in healthy and pathological aging, and so it is important to consider how ovarian hormones contribute to either process. This can advance sex-based sleep disorder identification and treatment approaches, while clarifying why women are at elevated risk for dementia. This review highlights many remaining questions about effects of ovarian hormone loss on sleep; clearer answers that will enhance health and quality of life in women are, frankly, long overdue.

## Abbreviations

AD: Alzheimer’s disease
CEE: conjugated equine estrogen
E2: 17β-estradiol
EEG: electroencephalography
EMG: electromyography
EOG: electrooculography
FSH: follicle-stimulating hormone
HT: hormone therapy
MPOA: medial preoptic area
NREM: non-rapid eye movement sleep
OVX: ovariectomized
P: progesterone
PSG: polysomnography
REM: rapid eye movement sleep
SCN: suprachiasmatic nucleus
SE: sleep efficiency
SWA: slow-wave activity
SWS: slow-wave sleep
VLPO: ventral lateral preoptic nucleus
WASO: waking after sleep onset

## Additional Information

***Disclosure Summary:*** The authors have nothing to disclose.

***Data Availability:*** Data sharing is not applicable to this article as no datasets were generated or analyzed during the current study.
