# Corrigendum to: “Role of Ovarian Hormones in the Modulation of Sleep in Females Across the Adult Lifespan”

**DOI:** 10.1210/endocr/bqab227

**Published:** 2021-11-17

In the above-named article by Brown AMC and Gervais NJ (*Endocrinology*. 2020, 161(9); doi: 10.1210/endocr/bqaa128), a funder of the study was left out of the acknowledgments.

The funding acknowledgements of this article should include:

Nicole Gervais and Alana Brown were also supported by the Laboratory of Cognitive Neuroscience, Gender, and Health, funded by the: Wilfred and Joyce Posluns Foundation; Women’s Brain Health Initiative; Ontario Brain Institute; and Canadian Institutes of Health Research Grants CAN WJP-150643 and CAN 163902.

doi: 10.1210/endocr/bqaa128

